# AST-001 Improves Social Deficits and Restores Dopamine Neuron Activity in a Mouse Model of Autism

**DOI:** 10.3390/biomedicines11123283

**Published:** 2023-12-12

**Authors:** Ki Bum Um, Soyoung Kwak, Sun-Ha Cheon, JuHyun Kim, Su-Kyeong Hwang

**Affiliations:** 1 Astrogen Inc., 440, Hyeoksin-daero, Dong-gu, Daegu 41072, Republic of Korea; kb_um@astrogen.co.kr (K.B.U.); ksy1432@astrogen.co.kr (S.K.);; 2 Department of Pediatrics, School of Medicine, Kyungpook National University, Daegu 41944, Republic of Korea

**Keywords:** ASD, social interaction, dopaminergic neuron, pacemaking, calcium-activated potassium channel, valproic acid

## Abstract

Autism spectrum disorder (ASD) is a complex neurodevelopmental disorder characterized by impaired social communication and social interaction, restricted and repetitive behavior, and interests. The core symptoms of ASD are associated with deficits in mesocorticolimbic dopamine pathways that project from the ventral tegmental area (VTA) to the nucleus accumbens (NAc) and medial prefrontal cortex (mPFC). AST-001 is an investigational product currently in a phase 3 clinical trial for treating the core symptoms of ASD, with L-serine as the API (active pharmaceutical ingredient). Because the causes of ASD are extremely heterogeneous, a single genetic ASD model cannot represent all autism models. In this paper, we used the VPA-exposed model, which is more general and widely used than a single genetic model, but this is also one of the animal models of autism. Herein, we conducted experiments to demonstrate the efficacy of AST-001 as L-Serine that alters the regulation of the firing rate in dopamine neurons by inhibiting small conductance Ca^2+^-activated K^+^ channels (SK channels). Through these actions, AST-001 improved sociability and social novelty by rescuing the intrinsic excitabilities of dopamine neurons in VPA-exposed ASD mouse models that showed ASD-related behavioral abnormalities. It is thought that this effect of improving social deficits in VPA-exposed ASD mouse models is due to AST-001 normalizing aberrant SK channel activities that slowed VTA dopamine neuron firing. Overall, these findings suggest that AST-001 may be a potential therapeutic agent for ASD patients, and that its mechanism of action may involve the regulation of dopamine neuron activity and the improvement of social interaction.

## 1. Introduction

Autism spectrum disorder (ASD) is a highly heterogeneous group of neurodevelopmental disorders characterized by impaired social interaction and communication, restricted interests, and repetitive behaviors [1,2]. One in 36 (2.8%) children have been diagnosed with ASD, according to a recent report from the Centers for Disease Control and Prevention [3,4].

While a precise cause has not been established, many studies suggest that autism develops from a combination of genetic and nongenetic, or environmental, components that impact brain development [5]. These various causes can lead to dopamine dysfunction, which plays an important role in social deficits, and is a core symptom of autism [6,7,8,9,10]. Among multisynaptic neural circuits, the mesocorticolimbic dopamine pathway projecting from the ventral tegmental area (VTA) to the medial prefrontal cortex (mPFC) or nucleus accumbens (NAc) regulates social cognition and social reward [6,7]. For example, mutations in *SHANK3* or *NLGN3*, synaptic function genes, result in abnormal dopamine pathways and social deficits [8,9]. Abnormal dopamine pathways can lead to an imbalance in dopamine levels determined by the rate of spontaneous firing, and decreased dopamine levels in mesolimbic dopamine pathways impair social interactions [10].

Valproic acid (VPA) is an antiepileptic drug that induces offspring with ASD when exposed during gestation, which is a critical period for embryonic brain development [11]. Since the VPA-exposed model in rodents shows a similarity of core symptom behaviors to human ASD patients, it is frequently used as an animal model study of ASD [12]. A reduction in the dopamine pathway has been reported in the VPA rodent models [13]. It is also well known that the function of voltage-dependent or -independent ion channels is altered in ASD models [14,15]. Among them, small conductance Ca^2+^-activated K^+^ channels (SK channels) are known to be pacemaker ion channels that determine spontaneous firing rates by regulating afterhyperpolarization (AHP) in dopamine neurons [16,17]. It has been reported that the aberrant activity of SK channels exists in patient and animal models of ASD [18]. A decrease in intrinsic excitability by SK channel overexpression in *PTEN* haploinsufficiency-associated autism mice has been reported [18]. Therefore, the modulation of the spontaneous firing of dopamine neurons via pacemaker ion channels may be the key to improving social impairments in ASD.

AST-001 is an L-isomer of serine (L-Serine, patents related to AST-001 include Korean Patent No. 2091620 and PCT/KR2022/003789) [19], a conditionally essential amino acid for the brain, because it acts as a neurotrophic factor, including neuronal signaling, neuroprotection, development, and proliferation [20]. L-Serine is known to be effective in neurological diseases such as epilepsy, schizophrenia, and Alzheimer’s Disease [21,22,23]. In a previous study, we demonstrated the neuroprotective effect of L-Serine against oxidative-stress-related apoptotic cell death through glutathione biosynthesis, suggesting the potential of L-Serine as a treatment for neurodevelopmental diseases [24].

While there are U.S. Food and Drug Administration-approved treatments to improve repetitive behaviors, such as risperidone and aripiprazole [25], there are no effective treatments to improve social interaction, a core symptom of ASD.

In this study, we found that the acute application of AST-001, as an L-Serine, increased the spontaneous firing of dopamine neurons through SK channel inhibitions. In the chronic oral administration of AST-001, VPA-exposed ASD mouse models improved their levels of sociability and social novelty, and decreased their levels of anxiety. Furthermore, the chronic administration of AST-001 normalized spontaneous firing and SK channel current of dopamine neurons in VPA-exposed model mice. This study aimed to evaluate the effect of AST-001 on improving social deficits, a core symptom of ASD, through the intrinsic excitability of dopamine neurons through changes in specific ion channels.

## 2. Materials and Methods

### 2.1. Animals

All experiments on animals were carried out with the approved animal care and use guidelines of the Laboratory Animal Research Center in Astrogen Co., Ltd. (Daegu, Republic of Korea), and all the experimental protocols were approved by the Ethical Committee of Astrogen Inc. (ASTA2022-004, Daegu, Republic of Korea). To examine the acute cellular mechanism study for AST-001, 3–4-week-old C57BL/6N male mice were used. To perform the chronic administration of AST-001 study, 5–7-week-old C57BL/6N male mice were used for behavioral and electrophysiological tests. Mice were group-housed (2–6 per cage) in a vivarium that was controlled for humidity, temperature (21–23 °C), and photoperiod (12 light/dark cycles). Mice had ad libitum access to food and water throughout the experiment. In all experiments, we used male mice (postnatal weeks 3–7) weighing 9–25 g.

### 2.2. Chemicals

All salts and drugs were purchased from Sigma-Aldrich (St. Louis, MO, USA), except NBQX (Tocris, Cat. #: 1044), CPP (Tocris, Cat. #: 0247), SR 95531 (Tocris, Cat. #: 1262), and CGP-55845 (Tocris, Cat. #: 1248). AST-001 was synthesized at Tianjin Tianyao Pharmaceuticals (Tianjin, China). For acute slice recording, agonist or antagonist stock solutions were prepared in deionized water or DMSO, and stocks were diluted to final concentrations in external solutions.

### 2.3. Induction of VPA-Exposed ASD Mouse Model

VPA concentration and schedule were determined based on [12]. Female mice were mated with adult male mice of the same strain. Gestational days were determined by the presence of a vaginal plug on embryonic day 1. VPA sodium salt (Sigma-Aldrich, p4543) was dissolved in (0.9% NaCl saline) to a concentration of 600 mg/kg. On gestational day 12, VPA-dams received a single subcutaneous injection of 600 mg/kg VPA; control groups received a single injection of saline as vehicles (0.9% NaCl).

### 2.4. AST-001 Administration

AST-001 was dissolved in distilled water for in vivo experiments with per oral (p.o.) administration. VPA-exposed mice received a 2-week chronic treatment with AST-001 (500 mg/kg) or vehicle (saline) from postnatal day 21. The average weight of the vehicle group (*n* = 6) was 10.09 ± 0.62 g at postnatal day 21 (before the administration of AST-001), 16.22 ± 0.75 g at postnatal 28, and 20.43 ± 0.71 g at the start of the behavioral experiment (postnatal 36). The VPA group (*n* = 6) was 9.15 ± 0.38 g at postnatal day 21, 15.95 ± 0.75 g at postnatal day 28, and 19.55 ± 0.62 g at the start of the behavioral experiment (postnatal 36). The AST-001-treated group (*n* = 6) was 9.35 ± 1.04 g at postnatal day 21, 15.00 ± 1.28 g at postnatal day 28, and 19.1 ± 1.55 g at the start of the behavioral experiment (postnatal 36). Drugs were administered orally at 10 mL/kg per animal using an oral gavage.

### 2.5. Behavioral Tests

#### 2.5.1. Three-Chamber Sociability and Social Novelty Test

The size of the three-chamber cage was 20 cm (length) × 40.5 cm (width) × 22 cm (height). The sociability and social novelty tests were performed at 6 weeks of age in the control and VPA-exposed ASD mouse model. All mice in the tests were male mice of the same strain (C57BL/6N) and the same age (P35). Tests consisted of three sessions. The first session allowed the mouse to freely move about an empty three-chamber cage for 10 min for habituation. The next session tested sociability; the mouse was placed in the center chamber while a stranger mouse and an empty cage (object) were placed on each side of the chamber. During the sociability test session, preference was measured using the sniffing time of each cage for 10 min. After sociability tests, social novelty tests were performed. In this session, familiar and non-familiar mice were placed on each side chamber. Before the tests, the subject mouse was again gently placed in the center chamber while the object was replaced with a new stranger mouse [26]. The sociability and social novelty preference index was calculated using the following equation: sociability test = (spent time stranger – spent time empty)/(spent time stranger + spent time empty), social novelty preference test = (spent time non-familiar – spent time familiar)/(spent time non-familiar + spent time familiar).

#### 2.5.2. Elevated Plus Maze (EPM) Test

Anxiety was examined using the elevated plus maze tests. Four arms (length: 75 cm; width: 5 cm) were connected and elevated to a height of (50 cm) from the floor. Mice were placed on the central platform facing an open arm and allowed to explore the maze for 5 min. Then, time spent in the open arm and entries into the open or closed arms was recorded. The arm entry was defined as 50% of the body being positioned within the arm.

### 2.6. Electrophysiology

#### 2.6.1. Midbrain Slice Preparation

All electrophysiological experiments were conducted according to Um et al., 2021 [27] with modifications. For slice preparations, mice were anesthetized with CO_2_ gas, and brains were quickly removed and horizontally sliced at 250 μm thickness by using a VT-1200 vibratome (Leica, Wetzlar, Germany) with an ice-cold cutting solution (in mM: 25 NaHCO_3_, 2.5 KCl, 1.25 NaH_2_PO_4_, 0.5 CaCl_2_, 7 MgSO_4_, 215 Sucrose, and 25 D-glucose, pH 7.3 oxygenated with 95/5% O_2_/CO_2_). After the preparation of slices, the slices were incubated at 33 °C in a holding solution (in mM: 92 NaCl, 30 NaHCO_3_, 3 KCl, 1.2 NaH_2_PO_4_, 5 Sodium ascorbate, 2 CaCl_2_, 1.5 MgSO_4_, and 25 D-glucose, pH 7.3 oxygenated with 95/5% O_2_/CO_2_). Slices were kept at 33 °C in a holding solution for 30 min to 5 h before being used for recording.

#### 2.6.2. Patch Clamp Recording

Sliced tissues were transferred to a submerged slice chamber within a continuously perfusing oxygenated (95/5% O_2_/CO_2_ gas) artificial cerebrospinal fluid (aCSF in mM: 125 NaCl, 25 NaHCO_3_, 2.5 KCl, 1.25 NaH_2_PO_4_, 0.4 Sodium ascorbate, 2 CaCl_2_, 1 MgCl_2_, and 25 D-glucose, pH 7.3 oxygenated with 95/5% O_2_/CO_2_) at 33 °C. To exclude Glutamate and GABA synaptic inputs in slice recordings, glutamate receptor blockers (NBQX, AMPA receptor antagonist, Tocris, 1044; CPP, NMDA receptor antagonist, Tocris, 0247) and GABA receptor blockers (SR-95531, GABA_A_ receptor antagonist, Tocris, 1262; 1 μM CGP-55845, GABA_B_ receptor antagonist, Tocris, 1248) were included in the recording solutions. Dopamine neurons were visualized with a Prime BSI CMOS camera (Photometrics, Tucson, AZ, USA) on the ECLIPSE F1 microscope equipped with a Fluor DIC 40× lens (Nikon, Tokyo, Japan). To ensure the drug delivery for target neurons, cell bodies were selected around 50–100 μm from the surface of tissues. Conventional tight-seal (>3 GΩ) whole-cell patch-clamp recordings were performed using potassium-based internal solutions (in mM: 115 K-MeSO_4_, 20 KCl, 1 MgCl_2_, 10 HEPES, 2 Mg-ATP, 0.2 Na-GTP, 0.025 EGTA, 10 Na_2_-phosphocreatine, pH adjusted to 7.3 with KCl and osmolarity about 280~290 mOsm/kg). Voltage recordings were collected with a Multiclamp 700B amplifier and digitized with a Digidata 1440A (Molecular Devices, San Jose, CA, USA), low pass filtered at 2 kHz and digitized at 10 kHz. Seal resistance was monitored by a 100 ms test pulse of 10 mV and reported voltages were corrected for a liquid junction potential of ~−15–−20 mV between internal and external solutions which was measured using a flowing 3 M KCl electrode.

### 2.7. Immunostaining

For traces of recorded neurons, the internal patch solution contained biocytin (0.4%, *w*/*v*). After patch recording, brain slices were fixed with 4% paraformaldehyde in ice-cold 1× phosphate-buffered saline (in mM: 137 NaCl, 10 Na_2_HPO_4_, KH_2_PO_4_, pH adjusted to 7.4 with NaOH). Fixed slices were permeabilized with 0.1% Triton-X 100 in 2% normal goat serum solutions of PBS. After the rinsing of slices with 2% goat serum, slices were incubated with the primary antibody (mouse anti-TH, 1:000, MAB318, Millipore, Burlington, MA, USA) in 2% goat serum solution for 18 h at 4 °C. After being washed, streptavidin-594 (1:2000, Thermo Fisher, S11227, Waltham, MA, USA) was treated at room temperature for 2 h. After the antibody reaction, slices were rinsed again with 2% NGS solution. Finally, samples were acquired using a Cytation 5 cell imaging multimode reader (BioTek, Houston, TX, USA).

### 2.8. Quantification and Statistical Analysis

Electrophysiological data were analyzed using Clampfit (Molecular Devices, San Jose, CA, USA) and Igor Pro (Wavemetrics, Portland, OR, USA). All collected data were statically analyzed using Prism 9.3.1 software (GraphPad Software, San Diego, CA, USA). The spontaneous firing frequency was measured as the average value from 20 s of each condition. For graphical illustrations, CorelDraw^®^ 2019 software (Corel Corporation, Ottawa, ON, Canada) was used. All numeric data are presented as mean ± standard error of the mean (S.E.M.) or standard deviation (S.D.). Data were summarized as box plots, with the centerline showing the median, the top, and bottom of the box indicating the 25–75% range, and the whisker representing the 5–95% range. For comparison of data, paired/unpaired T-tests or analysis of variance (ANOVA) tests were performed to assess the statistical significance of the difference between groups, and after followed by Dunnett, Tukey, or Sidak post hoc tests. Dunnett post hoc tests were performed to compare different sample sizes between control, VPA, and AST-001-treated groups. Tukey post hoc tests were performed to compare the paired samples in an acute drug treatment study. Sidak post hoc tests were performd to compare multiple comparisons of the Two-way ANOVA test. *p*-values were shown to be significant at * *p*  <  0.05; ** *p*  <  0.01; *** *p*  <  0.001.

## 3. Results

### 3.1. AST-001 Increases Spontaneous Firing in Dopamine Neurons

**Figure 1:**
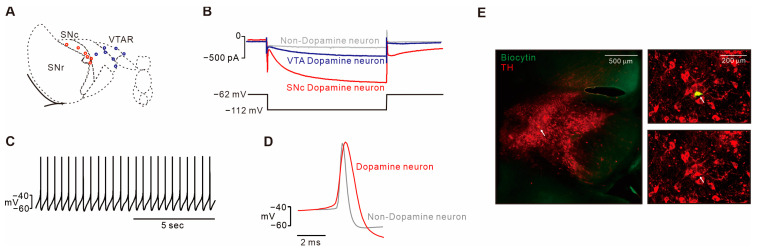
Identification of midbrain dopamine neurons. (**A**) Horizontal view of the midbrain showing anatomical segregation of functional subgroups. Circles showing the localization of individual recordings of SNc (red) or VTA (blue) dopamine neurons (3–4-week-old mice). (**B**) Representative trace showing the hyperpolarization-induced current (Ih) from SNc dopamine neurons (Red, >500 pA), VTA dopamine neurons (blue, >200 pA), or non-dopaminergic neurons (gray, <200 pA). (**C**) Representative voltage trace showing the regular and slow firing of midbrain dopamine neurons. (**D**) Alignment of action potential from spontaneous firing between dopamine neurons (red) and non-dopaminergic neurons (gray). Dopamine neurons have a larger spike width of an action potential. (**E**) Representative immunostaining data showing that the recording neurons were identified as dopaminergic neurons. Recording neurons were filled with 0.2% biocytin (green) and colocalized (white arrow) with TH-positive neurons (red).

We used C57BL/6N male mice aged 3–4 weeks for dissecting acute horizontal midbrain slices. To determine the mechanism of action of AST-001 on dopamine neurons, we measured electrophysiological properties by using whole-cell patch-clamp recordings (Figure 1). Neurons were selected from the substantia nigra compacta (SNc) and lateral or intermedial VTA regions (Figure 1A). To identify the recording neurons as dopaminergic, a hyperpolarization-induced inward current (Ih) was evoked (Figure 1B). With Ih recording, dopamine neurons in the SNc (>500 pA) and VTA (>200 pA) were recorded separately. In these voltage recordings, we observed the regular and slow spontaneous firing of dopamine neurons (Figure 1C). Dopamine neurons have a broader action potential width than non-dopaminergic neurons (Figure 1D). Recording neurons filled with 0.2% biocytin were identified as dopaminergic through co-immunostaining with tyrosine hydroxylase (TH, red) and streptavidin (green) after the electrophysiological experiments (Figure 1E). To ensure that the drugs were delivered to the recording neurons, cells within 100 μm from the surface of the slices were selected for recording.

**Figure 2:**
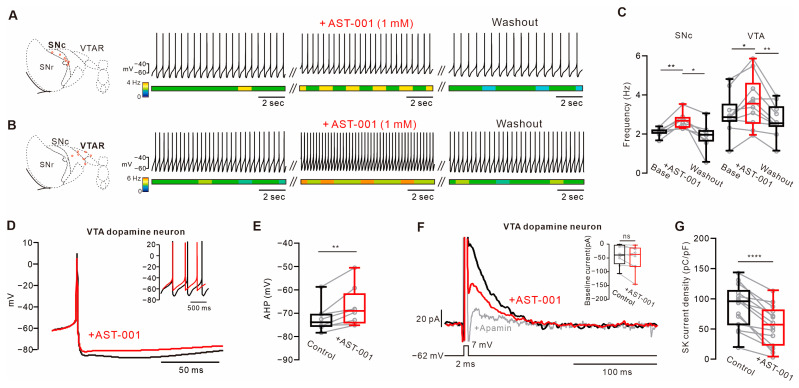
Acute treatment of AST-001 increases intrinsic excitability in midbrain dopamine neurons. (**A**,**B**) Red circles show the localization of individual recordings of SNc (**A**) and VTA (**B**) (3–4-week-old mice). Uncropped voltage traces are presented in Supplementary Figure S1. (**B**) Dopamine neurons (left). Representative voltage traces reveal the effect of AST-001 on spontaneous firing in the slice recording of SNc (**A**) and VTA (**B**) dopamine neurons (right). (**C**) Box plots summarize the changes in spontaneous firing through application of the AST-001 in dopamine neurons. (**D**) Trajectory trace presents the alignment of the AHPs between the control (black) and treated with AST-001 (red) of VTA dopamine neurons. (**E**) Box plots summarize AHPs of the spontaneous firing from (**B**). (**F**) Representative current traces show AP-evoked SK-channel-dependent outward tail currents (black = control, red = AST-001) of VTA dopamine neurons. (**G**) Box plots summarize the SK current density with or without AST-001 in dopamine neurons. (**C**) Analysis was performed with one-way ANOVA followed by Tukey’s post hoc test, and (**E**,**G**) were analyzed with paired-T tests. *p*-values were presented as in figure panels: * *p* < 0.05, ** *p* < 0.01, **** *p* < 0.0001, ns, not significant.

In acute administration of AST-001 (1 mM) via recording chamber perfusion for 10 min, a significant increase in spontaneous firing rate was observed in dopamine neurons from both SNc (Figure 2A,C, Base = 2.10 ± 0.22 Hz, +AST-001 = 2.68 ± 0.41, expressed as mean ± S.D., *n* = 8 from three mice, Supplementary Figure S1) and VTA (Figure 2B,C, Base = 3.01 ± 1.03 Hz, +AST-001 = 3.64 ± 1.3 Hz, expressed as mean ± S.D., *n* = 10 from four mice, Supplementary Figure S1) regions. Although the firing was changed by AST-001, the coefficient of variation was unchanged in both SNc and VTA dopamine neurons, suggesting that AST-001 had no effect on firing irregularity, such as a burst (Supplementary Figure S1C,D). After the washout of AST-001, firing rates were returned to baseline levels (SNc = 1.88 ± 0.69 Hz, VTA = 2.72 ± 0.84 Hz, expressed as mean ± S.D.). These observations suggest that AST-001 immediately increases the intrinsic excitability of dopamine neurons. 

### 3.2. AST-001 Alters Afterhyperpolarization through SK Channel Inhibition

In dopamine neurons, spontaneous firing frequency is determined by multiple pacemaker conductance [24]. To investigate which channels are altered by AST-001 to increase pacemaker activity, spontaneous action potential traces were aligned before and after the treatments with AST-001 (Figure 2D,E). As shown in Figure 2D, we observe that the AHP is suppressed by the treatment with AST-001 (V_AHP_, control = −72.10 ± 6.12 mV, +AST-001 = −67.35 ± 8.49 mV, expressed as mean ± S.D., *n* = 8 from 3 mice), suggesting that AST-001 increases spontaneous firing rates in dopamine neurons by altering the AHPs during interspike intervals.

In dopamine neurons, AHPs are regulated by SK channels [16,17]. Therefore, we investigated the effect of AST-001 on SK channels in dopamine neurons. To directly assess the ability of AST-001 to modulate SK channels, action potential (AP)-evoked SK-channel-dependent outward tail current was measured (Figure 2F,G). When AST-001 was acutely treated for 10 min, we observed that the treatment with AST-001 reduced the SK current (Figure 2G, I_SK_, control = 88.07 ± 35.90 pC/pF, +AST-001 = 56.04 ± 33.47 pC/pF, expressed as mean ± S.D., *n* = 14 from 6 mice). Additionally, AST-001 did not affect baseline current, showing that AST-001 did not induce other ion fluxes during AP-evoked current induction (Figure 2F, inset, control = −40.48 ± 37.70pA, +AST-001 = −51.54 ± 47.75 pA, *n* = 8 from 4 mice).

From these results, we suggest that AST-001 increases the firing rate by inhibiting SK channel inhibitions.

### 3.3. AST-001 Improves Social Interaction in ASD Mouse Models

**Figure 3:**
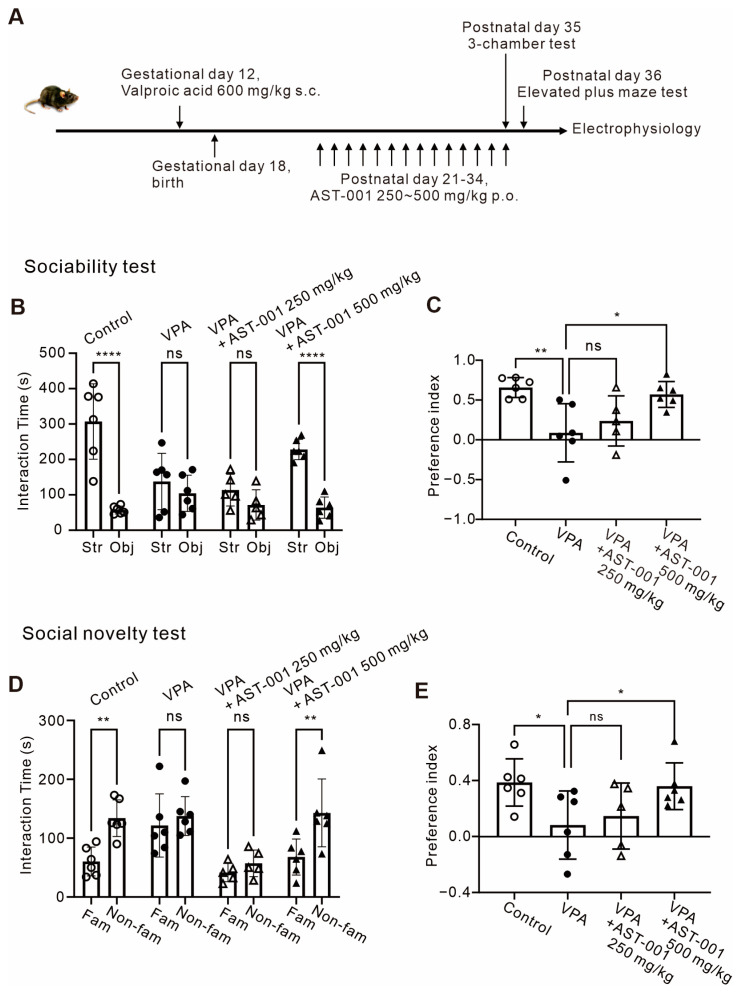
AST-001 improves social interaction in the VPA-exposed ASD mouse model. (**A**) The timeline of experiments. (**B**) Histograms showing the interaction time (sec) in the sociability test (5–6-week-old mice). (**C**) Histogram showing the sociability preference index. (**D**) Histogram showing the interaction time (sec) in the social novelty test (5–6-week-old mice). (**E**) Histogram showing the social novelty preference. All data are expressed as mean ± SD, (**B**,**D**) analysis was performed using two-way ANOVA followed by Sidak’s post hoc test, (**C**,**E**) analysis was performed using one-way ANOVA followed by Dunnett’s post hoc test. *p*-values were presented as in figure panels: * *p* < 0.05, ** *p* < 0.01, **** *p* < 0.0001, ns, not significant.

To confirm the therapeutic effect of AST-001 in social impairments, a core symptom in the animal model of ASD, we used the VPA-exposed ASD mouse model (Figure 3). VPA-exposed model mice were generated using a subcutaneous injection of a single dose of VPA (600 mg/kg) to pregnant C57BL6/N mice on gestational day 12 (Figure 3A). To evaluate the efficacy of AST-001 on autistic-like behavior, AST-001 was orally administered to VPA-exposed model mice on postnatal day 21 for 2 weeks, and 3-chamber sociability and social novelty tests were performed (Figure 3A). The dosage was referenced from our previous pharmacokinetics studies of AST-001 in healthy human subjects [19].

Sociability is measured using the preference between strangers and objects (Figure 3B,C). As shown in Figure 3B, VPA-exposed model mice lost their preference for either conspecific stranger mice or empty wire cages (objects), whereas the control group tended to approach stranger mice (interaction time, control, *n* = 6, obj = 57.17 ± 11.02 s, str = 307.17 ± 106.52 s; VPA, *n* = 6, obj = 104.5 ± 51.27 s, str = 137.6 ± 79.68; expressed as mean ± S.D.; Figure 3B). Oral administration of AST-001 (500 mg/kg) in VPA-exposed model mice displayed an improvement in sociability due to an increased preference for stranger mice similar to control groups (interaction time, VPA + AST-001 250 mg/kg, *n* = 5, obj = 113.4 s, str = 113.4 s, VPA + AST-001 500 mg/kg, *n* = 6, obj = 64.0 s, str = 228.1 s; expressed as mean ± S.D.; Figure 3B; preference index, control = 0.65 ± 0.12, *n* = 6; VPA = 0.08 ± 0.36, *n* = 6; VPA + AST-001 250 mg/kg = 0.23 ± 0.31, *n* = 5; VPA + AST-001 500 mg/kg = 0.57 ± 0.16, *n* = 6; expressed as mean ± S.D.; Figure 3C).

In social novelty tests, VPA-exposed model mice displayed no significant social preference between familiar and non-familiar conspecifics compared to control groups (interaction time, control, *n* = 6, fam = 60.3 s, non-fam = 134.0 s; VPA, *n* = 6, fam = 121.6 s, non-fam = 137.6 s; expressed as mean ± S.D.; Figure 3D; preference index, control = 0.39 ± 0.17, *n* = 6; VPA = 0.08 ± 0.24, *n* = 6; expressed as mean ± S.D.; Figure 3E). VPA-exposed model mice treated with 250 mg/kg of AST-001 had no significant change in preference (interaction time, VPA + 250 mg/kg, *n* = 5, fam = 41.7 s, non-fam = 57.34 s, Figure 3D; preference index, VPA + AST-001 250 mg/kg = 0.15 ± 0.24, *n* = 5, Figure 3E), whereas those treated with 500 mg/kg of AST-001 showed a significant increase in social preference for the non-familiar conspecifics (interaction time, VPA + 500 mg/kg, *n* = 6, fam = 68.0 s, non-fam = 142.9 s, Figure 3D; preference index, VPA + AST-001 500 mg/kg = 0.36 ± 0.16, *n* = 6, Figure 3E). Altogether, our findings suggest that AST-001 improves deficits in the social interaction of VPA-exposed model mice.

### 3.4. AST-001 Improves Anxiety in ASD Mouse Models

**Figure 4:**
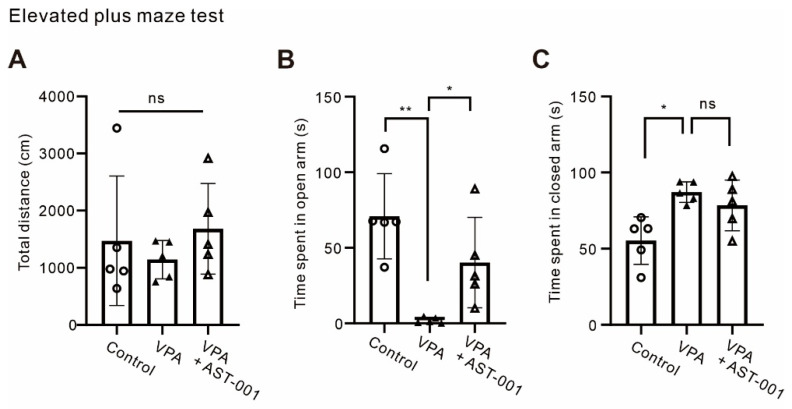
AST-001 ameliorated anxiety-like behaviors in the VPA-exposed ASD mouse model. (**A**) Histograms showing the total distance of movements in a plus maze of control, VPA-exposed mice, and VPA-exposed mice with AST-001 (5–6-week-old mice). (**B**,**C**) Histograms showing the restoration of anxiety levels in VPA-exposed mice through the oral administration of AST-001 (5–6-week-old mice). All data are expressed as mean ± S.D. Analysis was performed using one-way ANOVA followed by Dunnett’s post hoc test. *p*-values were presented as in figure panels: * *p* < 0.05, ** *p* < 0.01, ns, not significant.

It is known that patients with ASD usually also have anxiety [28]. Therefore, we performed an elevated plus maze (EPM) test to confirm the efficacy of AST-001 on anxiety in VPA-exposed model mice (Figure 4). In this test, the anxiety level was measured by time spent in the open arm (total distance, Figure 4A; time spent in open arm, Figure 4B, *n* = 5; time spent in closed arm, *n* = 5, Figure 4C). VPA-exposed model mice showed a significantly reduced dwell time spent in the open arm compared to control groups (control = 70.96 ± 28.22 s, VPA = 2.06 ± 1.96 s, expressed as mean ± S.D., Figure 4B). The administration of AST-001 to VPA-exposed model mice showed less anxiety-like behavior compared to the VPA-exposed model group (VPA + AST-001 = 40.29 ± 29.95 s, Figure 4C). Our results suggest that the administration of AST-001 ameliorates social deficits and anxiety-like behavior in VPA-exposed model mice.

### 3.5. Chronic AST-001 Normalizes Intrinsic Excitability in ASD Mouse Models

**Figure 5:**
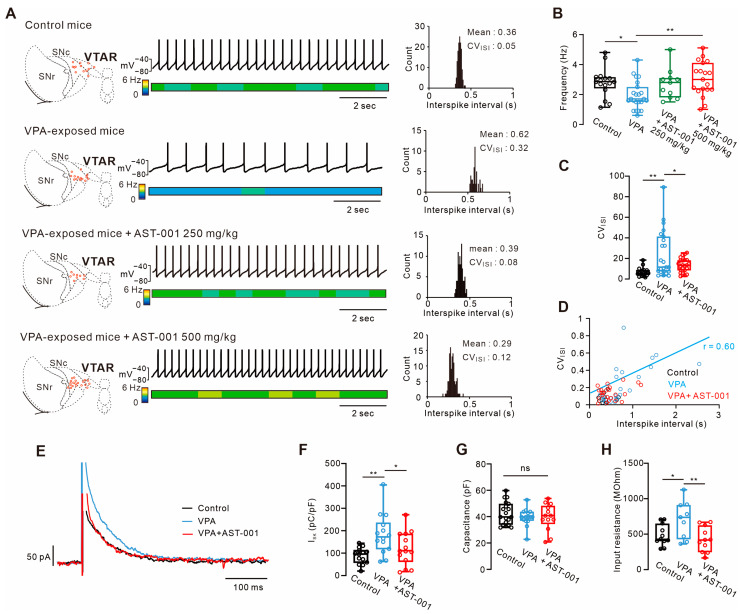
AST-001 rescues the intrinsic excitability in the VPA-exposed ASD mouse model. (**A**) Midbrain diagrams showing the localization of individual dopamine neuron recordings (red circles, left, 5–7-week-old mice). Representative voltage traces showing the spontaneous firing of VTA dopamine neurons in control mice, VPA-exposed mice, or VPA-exposed mice with oral administration of AST-001 (250 or 500 mg/kg, center). Histogram from 40 s of data from the firing recording of each group (right). (**B**) Box plots summarizing the spontaneous firing frequency in control, VPA-exposed mice, or VPA-exposed mice with AST-001 at 250 or 500 mg/kg. (**C**) Box plots showing the coefficient of variation (CV) from interspike intervals of spontaneous firing recording from control, VPA, and VPA + AST-001 (500 mg/kg). (**D**) Histogram graph showing the correlation of between interspike intervals and coefficient of variation of interspike intervals from control, VPA, and VPA + AST-001 (r = correlation coefficient). (**E**) Representative traces showing the AP-evoked SK-channel-dependent outward tail current from control, VPA, and VPA + AST-001. (**F**) Box plots summarizing the AP-evoked SK current (5–7-week-old mice). (**G**) Box plots showing the membrane capacitance (pF) of control, VPA-exposed mice, or VPA-exposed mice with AST-001. (**H**) Box plots showing the input resistance (MOhm) of control, VPA-exposed mice, or VPA-exposed mice with AST-001. All analyses were performed using one-way ANOVA followed by Dunnett’s post hoc test. *p*-values were presented as in figure panels: * *p* < 0.05, ** *p* < 0.01, ns, not significant.

To determine whether improvements in social behavior and anxiety induced by AST-001 were related to neuronal excitability, changes in the spontaneous firing rate of dopamine neurons due to AST-001 administration were observed with the administration of AST-001 (Figure 5). VPA-exposed model mice had a significantly slower spontaneous firing rate than control mice (control = 2.82 ± 0.97 Hz, *n* = 16 from 10 mice; VPA = 1.93 ± 0.93 Hz, *n* = 23 from 11 mice, expressed as mean ± S.D., Figure 5A). Moreover, the coefficient of variation from interspike intervals of the spontaneous firing showed that VPA-exposed model mice had significantly increased firing irregularity compared to control mice (Coefficient of variation, control = 0.06 ± 0.04, *n* = 16, VPA = 0.24 ± 0.22, *n* = 23; expressed as mean ± S.D.; Figure 5C). In VPA-exposed mice, the coefficient of variation showed a correlation with the interspike intervals (r = 0.6, Figure 5D), suggesting that irregularity increases as firing becomes slower. In these mice, the oral administration of AST-001 (500 mg/kg) for 2 weeks restored spontaneous firing rates similar to control mice (+AST-001 500 mg/kg = 3.04 ± 1.14 Hz, expressed as mean ± S.D, *n* = 19 from five mice), whereas 250 mg/kg of AST had no significant change (+AST-001 250 mg/kg = 2.89 ± 1.14 Hz, expressed as mean ± S.D., *n* = 11 from three mice). In addition, it was also observed that the firing irregularity returned to the control mice level (coefficient of variation, +AST-001 = 0.13 ± 0.06, expressed as mean ± S.D., *n* = 23; Figure 5C).

We observed intrinsic excitability-related pacemaking ion channels, such as SK channels. In ap-evoked SK-channel-mediated tail current, VPA-exposed mice showed more hyperactive tail current than controls in VTA dopamine neurons (I_SK_, control = 88.08 ± 35.90 pC/pF, expressed as mean ± S.D., *n* = 14 from five mice; VPA = 183.48 ± 90.29 pC/pF, expressed as mean ± S.D., *n* = 14 from six mice; Figure 5E,F). In VPA-exposed mice, the chronic administration of AST-001 for 2 weeks rescued the SK channel current to control levels (I_SK_, VPA + AST-001 = 121.17 ± 73.85 pC/pF, expressed as mean ± S.D., *n* = 14 from six mice; Figure 5E,F). There was no significant difference in cell capacitance between control, VPA-exposed mice, and VPA-exposed model mice with AST-001 (control = 42.74 ± 8.64 pF, expressed as mean ± S.D., *n* = 19 from 10 mice; VPA = 39.80 ± 6.52 pF, *n* = 15 from 11 mice; VPA + AST-001 = 39.77 ± 10.11 pF, expressed as mean ± S.D., *n* = 13 from 4 mice; Figure 5G). Altogether, we suggest that the reduced firing rates in dopamine neurons from VPA-exposed mice are associated with increments in SK channel conductance, and that chronic treatment with AST-001 restores these changes.

## 4. Discussion

In this study, we investigated the mechanisms through which L-Serine, named AST-001, alters the intrinsic excitability of dopamine neurons by inhibiting SK channels. AST-001 showed potential as a drug for treating the core symptoms of ASD because it normalized the intrinsic excitability of dopamine neurons through SK channels and improved social dysfunction. 

ASD is a neurodevelopmental disorder caused by a combination of various genetic and environmental factors [5]. Ion channels are critical to the cellular function in neurons because they receive and transmit intercellular signals by regulating ion flux [29]. Therefore, channelopathies may have a significant impact on brain function and may serve as important targets in ASD. Recent studies have shown that genetic variations in Ca^2+^, Na^+^, and K^+^ channels contribute not only to ASD, but also to bipolar disorder, schizophrenia, and other neuropsychiatric disorders [30]. In the CNS regions, ASD exhibits hyper- or hypo-active neural excitability due to abnormal ion channel functions that induce an imbalance of the synaptic transmission of the underlying neural networks [14]. Several studies have reported dysfunction of the dopamine pathway in ASD models, which is mainly caused by a decrease in dopamine release due to the hypo-excitability of dopamine neurons [31]. Therefore, the regulation of the firing of the dopamine neuron is important for the treatment of ASD. 

Our electrophysiological study showed that the acute bath application of AST-001 altered intrinsic firing activity by inhibiting SK channel currents. SK channels are Ca^2+^-activated K^+^ channels that underlie AHP and thus regulate firing frequency in dopamine neurons. Firing regulation in the dopamine neuron is maintained through robustly organized multiple ion channels that act in slow depolarization which consists of three phases: AHP, steady depolarization, and acceleration to the threshold during interspike intervals [27]. As shown in Figure 2, AST-001 reduced the AHP by inhibiting SK channels, resulting in a shorter duration of interspike intervals that increased spontaneous firing rates. Therefore, AST-001 may increase dopamine release through SK channel inhibition.

We confirmed that AST-001 partially inhibits AP-evoked SK-channel-mediated current, but could not confirm the detailed mechanism of how it inhibits SK channel current. There are three hypotheses on how AST-001 inhibits the SK channels. First, AST-001 may bind directly to the SK channel. In this hypothesis, conformational changes through the specific or non-specific binding of AST-001 may suppress the channel current. However, the binding pocket involved in antagonisms of SK channels is still unknown. The next possibility is the regulation of SK channels through changes in membrane potential. AST-001, as an L-Serine, can depolarize the membrane potential through Na^+^ influx through SNAT (sodium-coupled neutral amino acid transporter) [32]. Moreover, the production of glycine can cause the hyperpolarization of the membrane potential through the influx of Cl^−^ through the glycine receptor [33]. Changes in membrane potential affect the intracellular Ca^2+^ dynamics through voltage-dependent Ca^2+^ channels. However, our study confirmed that 1 mM AST-001 did not change the basal current during voltage-clamp mode held at—60 mV (Figure 2F, inset histogram). These data suggest that AST-001 does not induce the influx of cations or Cl^−^. A last possibility is the regulation of SK channel activity through intracellular phosphorylation pathways. It is known that the activity of SK channels in dopamine neurons can be modulated by the phosphokinase A pathway [34]. However, there is still no evidence that L-Serine is involved in this pathway.

Social impairment in ASD results from the reward circuitry dysfunction of the mesocorticolimbic dopamine pathway [31]. The hypo-function of the reward system through reduced dopamine release to the mPFC or NAc results in a lack of social reward-related interactions [31]. Since the concentration of dopamine released to the target region depends on the firing rate of dopamine neurons, improving social behaviors through regulating the firing rate of dopamine neurons may be the key to treating ASD.

We displayed, for the first time, that VPA-exposed model mice had slower spontaneous firing of the VTA dopamine neurons than normal mice. In rodent ASD pathophysiology, VPA is known to have multiple pharmacological effects [12]. VPA is an inhibitor of non-specific histone deacetylase (HDAC) that causes epigenetic changes during neurodevelopment [12]. Epigenetic modification causes the alteration of several neurodevelopmental factor expression patterns, such as pax6, ngn2, tbe2, and NeuroD1, resulting in an E/I imbalance in the neural network [35]. Moreover, the inhibition of glutathione biosynthesis due to the disruption of the methionine cycle causes fetal toxicity through oxidative stress in cells [35]. Additionally, it can cause maternal immune defects and interfere with neurodevelopment [12]. These effects of VPA in pregnancy contribute to deficits during a critical period of adolescent neurodevelopment and lead to the core symptoms of ASD, such as social impairments and repetitive behavior [13]. In previous studies, it was discovered that the dopamine pathway was weakened in VPA-exposed mice [13], but the detailed physiological cause has not been identified. Decreased firing rates lead to a reduced release of dopamine in axon terminals [13,36], which can be expected to lead to impaired social behavior. In SK channel recordings, we observed a larger current in VPA-exposed mice than in normal mice, suggesting that hyperactive SK channels induce the slower firing of dopamine neurons. Surprisingly, the chronic administration of AST-001 normalized the spontaneous firing rate of dopaminergic neurons in VPA-exposed mice, suggesting that this alteration was caused by normalizing the hyperactive SK channel current in VPA-exposed mice to normal levels. These results suggest that AST-001 improves social impairment related to abnormal dopamine pathways by restoring dopamine firing. Additionally, we observed social improvement and normalizing effects on dopaminergic neurons through the administration of AST-001 using only one ASD model induced by VPA. Therefore, to further reveal its efficacy as an ASD treatment, further experiments will be needed in other models such as Shank 3 or neuroligin 3 mutation mice [8,9], which reported dopamine dysfunctions, or chromosomal mutant ASD models. 

People with ASD are known to have problems with emotional and anxiety regulation, as well as social deficits. One study found that 80% of individuals with ASD reported very high levels of anxiety [37]. Patients with ASD with high anxiety may be difficult to treat and may have poor treatment outcomes. In EPM tests, our data confirmed that VPA-exposed mice had very high anxiety compared to the normal group, and when treated with AST-001, anxiety was confirmed to be reduced.

The limitations of this study include the inability to experimentally demonstrate a direct correlation between social interaction and the tonic firing of dopamine neurons. In this study, electrophysiological property-recorded neurons were located in lateral or intermediate regions of the VTA, where axons are mainly projected to the NAc [38]. Several studies have shown that optogenetic and chemogenic activation of VTA-NAc dopamine pathways improved social interactions in the ASD mouse model, whereas the inactivation of these pathways caused the impairment of social interactions in normal mice [8,9,10], suggesting that the mesolimbic dopamine pathway is a key driver of social interactions in ASD. Therefore, future studies will elucidate the relationship between behavioral changes induced through treatment with AST-001 in social interaction and the tonic firing of dopamine neurons.

**Figure 6:**
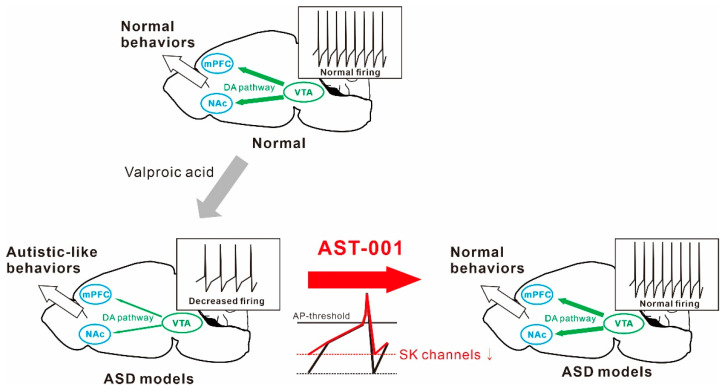
Schematic diagrams illustrate the therapeutic efficacy of AST-001 on the valproic acid (VPA)-exposed autism spectrum disorder (ASD) mouse model.

In conclusion, our results demonstrated that the administration of AST-001 restored the spontaneous firing rate through the normalization of SK channel activity in VTA dopamine neurons in mice exposed to VPA, thereby improving social interaction (Figure 6). These results suggest that the spontaneous firing of dopamine neurons may be a cellular mechanism that underpins the development of social interactions. Our findings indicate AST-001 to be a promising candidate drug for treating the core symptoms of ASD.

## Data Availability

The data that support the findings of this study are available on request from the corresponding author.

## Supplementary Materials

The following are available online at https://www.mdpi.com/article/10.3390/biomedicines11123283/s1, Figure S1: Acute treatment of AST-001 increases spontaneous firing rate but does not alter irregularity in midbrain dopamine neurons.
