# Supplementary material for: AST-001 Improves Social Deficits and Restores Dopamine Neuron Activity in a Mouse Model of Autism
Source: Biomedicines. 2023 Dec 12;11(12):3283. doi: 10.3390/biomedicines11123283 (PMC10741043; doi:10.3390/biomedicines11123283)
Supplement: Supplementary file 1 [file biomedicines-11-03283-s001.zip › biomedicines-2718109-supplementary.pdf]

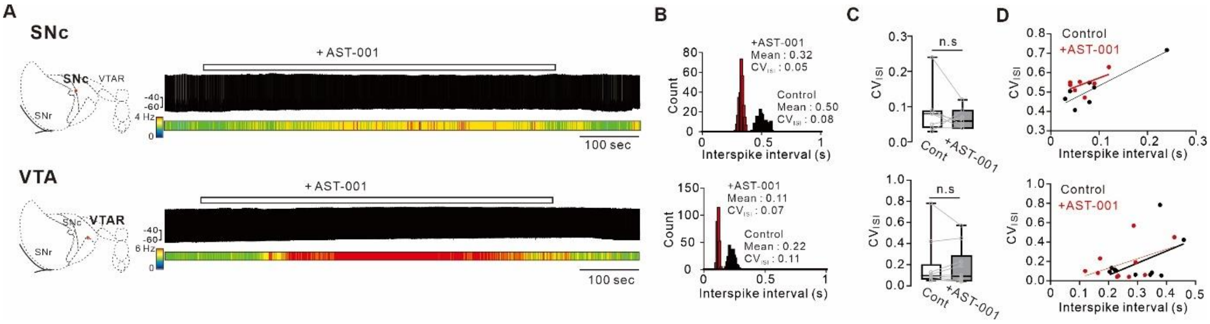

Supplementary Figure S1. Acute treatment of AST-001 increases spontaneous firing rate but does not alter irregularity in midbrain dopamine neurons. (**A**) Diagram and voltage trace showing the whole trace of a single neuron recording (right) during acute treatment with AST-001 in SNc (top) or VTA (bottom) dopamine neurons. recording neuron is marked by recircle in diagram (left). (**B**) Histogram showing interspike interval from (A). (**C**) box plots showing the summarize coefficients of variation from interspike interval during the before and after treatment of AST-001 of Figure2A, B. (**D**) Histogram showing the correlation between interspike interval and coefficients of variation of interspike interval. (C) analysis was performed by paired-T tests. p-values were presented as in figure panels: ns, not significant.
